# Development of Polymeric Micelles of Oleanolic Acid and Evaluation of Their Clinical Efficacy

**DOI:** 10.1186/s11671-020-03348-3

**Published:** 2020-06-22

**Authors:** Joo Young An, Hee Seon Yang, Na Rae Park, Tae-sung Koo, Bungchul Shin, Eun Hee Lee, Sun Hang Cho

**Affiliations:** 1 Department of Bio and Drug Discovery Division, Innovative Target Research Center, Korea Research Institute of Chemical Technology, 141 Gajeongro, Yuseong, Daejeon, 34114 Republic of Korea; 2 College of Pharmacy, Korea University Sejong Campus, 2511 Sejong-ro, Sejong City, 30019 Republic of Korea; 3 Graduate School of New Drug Discovery and Development, Chungnam National University, 99 Daehakro, Yusung, Daejeon, 34134 Republic of Korea

**Keywords:** Formulation, Stability, Polymers, Delivery, Penetration

## Abstract

Oleanolic acid has been used only as a subsidiary agent in cosmetic products. The aim of the study is to show the effect of oleanolic acid as an active ingredient for the alleviation of wrinkles in humans and to develop a polymeric micelle formulation that enables poorly soluble oleanolic acid to be used as a main ingredient in cosmetic products for reducing wrinkles. The solubility of oleanolic acid was evaluated in solubilizers, surfactants, and polymers. The particle sizes and shapes of polymeric micelles containing oleanolic acid were evaluated by electrophoretic light scattering spectrophotometer and scanning electron cryomicroscopy. Encapsulation efficiency and skin permeation were measured by HPLC. Stability of the polymeric micelles stored at 40 °C for 3 months was evaluated by visual observation, particle size measurement, and oleanolic acid content measurement. Polymeric micelles in final product ampoule form were applied around the eyes of 23 female subjects for 8 weeks. Five skin parameters were evaluated by optical profilometry every 4 weeks for 8 weeks. In addition, professionals made visual observations of the skin and a human skin irritation study was conducted. Polymeric micelles of oleanolic acid with a particle size of less than 100 nm were prepared using Capryol 90® and poloxamer. The skin permeation rate of the oleanolic acid in the polymeric micelles was higher than that in the other solutions made of oleanolic acid dispersed in 2 different surfactants. No significant changes in particle size, color, or oleanolic acid content were observed, and the polymeric micelles stored at 40 °C for 3 months did not undergo phase separation. After 8 weeks of application, skin irritation had not developed and all five parameters evaluated by optical profilometry as well as the visual evaluation scores were significantly improved. This study showed that the polymeric micelles of oleanolic acid prepared in this study were stable and effective at alleviating wrinkles in humans as the principal active ingredient. Based on these findings, it is expected that polymeric micelles of oleanolic acid can be widely used in cosmetic applications.

## Introduction

Skin aging includes sagging (laxity), thinning, and wrinkles. It can be accelerated by infection, smoking, UV light, trauma, hormonal imbalance, stress, and/or pro-oxidants such as hydrolases including elastinase or collagenase [1]. Reactive oxygen species or free radicals generated by the causes mentioned above damage neighboring cells and result in reduced skin elasticity and thinning [2, 3]. Especially, UV light is known to trigger the generation of reactive oxygen species, which damage membrane lipids, cellular proteins, and DNA, and thereby accelerate the development of expression wrinkles, freckles, and melasma [1–4]. Oleanolic acid is an effective component of natural plant origin extracted from several plant species and used as a major medical and cosmetic ingredient. It is also found in fruits such as apples or pears [5]. As a kind of hydroxy pentacyclic terpene, oleanolic acid was first isolated from olive (*Olea europaea*) leaves and is found widely in plants including East Asian swertia (*Eugenia jambos*) and yellow gentian (*Gentiana lutea*). It promotes anti-aging functions via the synthesis of not only pro-collagen, which is important for collagen synthesis, but also ceramides and filaggrin, and also by inhibiting the activity of MMP-1, an enzyme that breaks down proteins such as collagen [5, 6]. On the basis of these findings, it can be assumed that oleanolic acid has a double anti-aging effect by not only promoting collagen production but also preventing collagen degradation [7]. As a result, oleanolic acid is a very promising anti-aging ingredient for cosmetic products. However, the use of oleanolic acid in cosmetic products as the main ingredient is limited by its poor aqueous solubility; thus, only small amounts of oleanolic acid as part of an emulsified formulation have been used as a subsidiary ingredient in cosmetic products [8]. Its physicochemical properties related to skin absorption include its melting point, molecular weight, partition coefficient, and hydrophilicity. Its melting point is higher than 300 °C, indicating that it is a highly crystalline material. Highly crystalline materials require greater energy for dissolution, show poor bioavailability due to their limited solubility and are therefore poorly absorbed [9]. In addition, highly hydrophilic or lipophilic compounds, or compounds of high molecular weight are known to not easily permeate the skin [10, 11]. The most frequently used method of improving the skin permeation of such molecules is the synthesis of precursors or the use of colloidal drug carriers. In this regard, liposomes, emulsions, and polymeric micelles have been actively studied [12].

Polymeric micelles are self-assembled nano-scale aggregates forming core-shell structures in aqueous solution. Polymeric micelles are often made of di-block or tri-block copolymers which can form a hydrophobic inner core and hydrophilic outer shell [13, 14]. Polymeric micelles are considered more physically stable than surfactant micelles since the properties of polymeric micelles vary depending on the type and the ratio of polymeric monomers in a block copolymer and have relatively low critical micelle concentrations [15, 16].

In this study, we prepared polymeric micelles of oleanolic acid and evaluated their particle size and shape, and the resulting encapsulation efficiency and skin permeation rate of oleanolic acid. The physical stability of oleanolic acid in this form was also evaluated for 3 months. The human anti-wrinkle effect of oleanolic acid in actual cosmetic product formulation was also investigated.

## Materials and Methods

### Materials

Oleanolic acid, Tween 80, Tween 20, and Tween 60 was purchased from TCI (Tokyo, Japan). PEG 400, Pluronic F127, and Pluronic F68, were obtained from BASF (Ludwigshafen, Germany). Propylene glycol, PEG 300, and PEG 200 were obtained from JUNSEI (Tokyo, Japan). TRANSCUTOL P, LABRASOL, LAUROGLYCOL FCC, LABRAFAC, Capryol® 90, and Capryol™ PGMC were purchased from Gattefossé (Lyon, France). Disodium EDTA (Daejung Chemical & Metals Co., Ltd., Siheung, Korea), allantoin (Sigma Aldrich, St. Louis, MI, USA), dipropylene glycol (Daejung Chemical & Metals Co., Ltd., Siheung, Korea), propanediol (DuPont Tate & Lyle Bio Products Company, LLC, Loudon, USA), carbomer (The Lubrizol Corporation, Ohio, USA) PEG/PPG/polybutylene glycol-8/5/3 glycerin (NOF Corporation, Tokyo, Japan), sodium hyaluronate (TCI, Tokyo, Japan), beta-glucan (SK Bioland, Cheonan, Korea), phenoxyethanol (Daejung Chemical & Metals Co., Ltd., Siheung, Korea), caprylyl glycol (J TWO K BIO CO., Ltd., Cheongju, Korea), and ethylhexylglycerin (J TWO K BIO CO., Ltd., Cheongju, Korea) were used for preparing the cosmetic product containing oleanolic acid. HPLC grade acetonitrile was obtained from Burdick & Jackson (Muskegon, MI, USA). Triple distilled water was used, and other solvents and reagents were EP and GR grade. Crlori:SKH1-hr hairless female mice were purchased from OrientBio (Seongnam, Korea).

### HPLC Analysis

Oleanolic acid was analyzed using Shimadzu LC-30 series HPLC (Shimadzu Corporation, Kyoto, Japan). A Kromasil 100 C18 250 mm × 4.6 mm, 5 μm, analytical column (Teknokroma, Barcelona, Spain) was used at ambient temperature. The mobile phase consisted of acetonitrile and water (85:15, v/v), the flow rate was 1 mL/min, and the injection volume 10 μL. Oleanolic acid was analyzed at UV *λ* = 210 nm. All measurements were taken at ambient temperature [17].

### Solubility and Formulation Optimization Study

A measured amount of oleanolic acid was added to a solubilizer, stirred at 60 °C for 48 h, and sonicated for 5 min using an ultrasonic cleaner. The suspension was centrifuged at 2000 rpm using a Universal 320R centrifuge (Hettich, Tuttlingen, Germany), and the supernatant was then collected. Then the supernatant was filtered through a 0.45 μm PVDF membrane filter (Whatman, Kent, UK). The solubility of oleanolic acid in the corresponding solubilizer was estimated by subtracting the weight of remaining solids from the sum of the initial weights of the oleanolic acid and the solubilizer (Table 1).

**Table 1:** Solubility of oleanolic acid in various solvents

Solvent	Solubility (%)
LABRAFAC	0.248
TRANSCUTOL P	0.8
LABRASOL®	0.012
Capryol® 90	2.5
LAUROGLYCOL 90	1.0
Carbitol	0.045
Polyethylene glycol 400	0.05
Triacetin	0.4
Brij 30	0.002
Brij 92	0.18

**Fig. 1:**
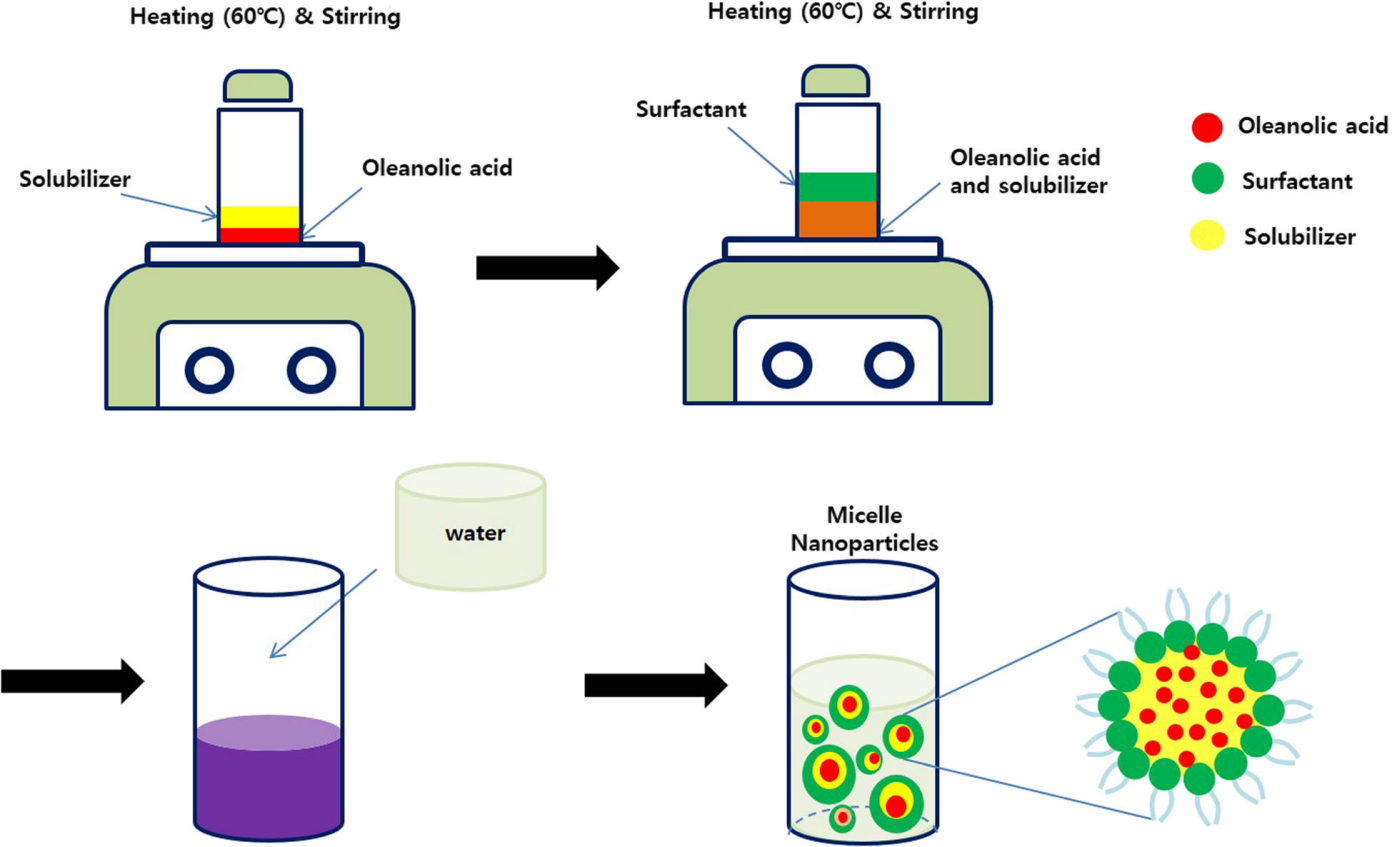
Schematic representation of preparing oleanolic acid micelles

The formulation optimization study was conducted using poloxamer 188, poloxamer 407, Tween 60, and Tween 80. Various ratios of amphiphilic polymers and surfactants were tried as summarized in Table 2. Each of oleanolic acid and Capryol® 90 was weighed, heated, and stirred at over 60 °C until clear solutions were observed. The polymers/surfactants shown in Table 2 were then added to the clear solution and stirred at over 60 °C until clear solutions were observed. The solutions were then dispersed in distilled water (Fig. 1). The resulting solution was left for approximately 48 h, and a visual inspection was then made to select the optimal formulation. The results of the visual inspections of each formulation in Table 2, including observations of precipitation, phase separation, transparency, and gelation, are summarized in Table 3.

**Table 2:** Compositions of micelles of oleanolic acid showing transparent liquid immediately after being diluted with distilled water

Composition (w/w%)	A	B	C	D	E	F	G	H	I	J	K	L
Oleanolic acid	0.05	0.05	0.05	0.05	0.05	0.05	0.05	0.05	0.05	0.05	0.05	0.05
Capryol 90	2	2	2	2	2	2	2	2	2	2	2	2
Poloxamer 188	6.5	7	10	-	-	-	-	-	-	-	-	-
Tween 80	-	-	-	7	8.5	10	-	-	-	-	-	-
Poloxamer 407	-	-	-	-	-	-	6	7	10	-	-	-
Tween 60	-	-	-	-	-	-	-	-	-	8	10	12

**Table 3:** Summary of visual observation of the polymeric micelles of oleanolic acid using the formulation shown in Table 2, 24 h after preparation

Composition (w/w%)	A	B	C	D	E	F	G	H	I	J	K	L
Phase state	PS	PS	PS	PS	PPT	PPT	T	T	G	PPT	PPT	PPT

*PS* phase separation, *PPT* precipitation, *T* transparent, *G* gelation

### Preparation of Polymeric Micelles of Oleanolic Acid

Polymeric micelles of oleanolic acid (PMO) were prepared using the formulations G and H in Table 2 using the method shown in Fig. 1. PMO-G and PMO-H were used for subsequent experiments and PMO-H in the cosmetic products for the clinical trials.

### Staining Test

Polymeric micellization when formulation G or formulation H was dispersed in water was confirmed by visual observation, i.e., transparency. PMO formation (PMO-G or PMO-H) was also confirmed by a stain test. Methylene blue was added to the mixtures of water and Capryol® 90, water, Capryol® 90, PMO-G, and PMO-H and the color of the solutions were visually observed and photographed.

### Particle Size Measurement

The ELS (electrophoretic light scattering) technique measures scattering intensity fluctuation from the particles as a function of time when the particles exhibit both random Brownian motion and oriented electrophoretic motion in a well-defined electric field. Particle electrophoretic mobility is measured by the ELS technique [18] and allowed the particle size of both PMO-G and PMO-H to be evaluated using an electrophoretic light scattering spectrophotometer (ELS-Z, Photal, Otsuka Electronics, Japan).

### Scanning Electron Cryomicroscopy (Cryo-SEM) Analysis

Cryogenic scanning electron microscopy or scanning electron cryomicroscopy (cryo-SEM) is a powerful technique for visualizing the state of the microstructure or nanostructure of colloidal polymer suspensions or dispersions after they have been immobilized by fast-freezing and fractured for imaging. The fracture is made and examined at − 196 °C, the normal boiling point of liquid nitrogen and far below the glass transition temperature of both bulk and fully coalesced particles. Cryo-SEM images reveal a range of responses of particles to fractures that propagate past them through ice [19]. Scanning electron cryomicroscopy (Tescan Mira 3 LMU FEG/Quorum Technologies PP3000T Cryo-SEM Sample Preparation System) was used to observe the shape of the PMOs.

### Encapsulation Efficiency

The encapsulation efficiency of the oleanolic acid polymeric micelles was evaluated. PMO was centrifuged at 2000 rpm for 15 min, and the supernatant was collected and analyzed by HPLC. The encapsulation efficiency was calculated as the amount of oleanolic acid in the polymeric micelles divided by the amount of oleanolic acid initially added (mg) during PMO preparation.

$$ \mathrm{Encapsulation}\ \mathrm{efficiency}\ \left(\%\right)=\kern0.37em \frac{\mathrm{Amount}\ \mathrm{of}\ \mathrm{oleanolic}\ \mathrm{acid}\ \mathrm{in}\ \mathrm{polymeric}\ \mathrm{micelle}\ \left(\mathrm{mg}\right)}{\mathrm{Amount}\ \mathrm{of}\ \mathrm{oleanolic}\ \mathrm{acid}\ \mathrm{in}\mathrm{itially}\ \mathrm{added}\ \left(\mathrm{mg}\right)}\times 100 $$

### Stability of PMO-H

The physical stability of PMO-H was evaluated by storing it at 40 °C for 3 months. Color changes, phase separation, the presence of precipitates, and turbidity changes were visually evaluated. PMO-H samples taken at regular time intervals were analyzed by HPLC to determine the amounts of oleanolic acid remaining and by an ELS-Z to determine the PMO-H particle size. The results are presented in Fig. 9

### In Vitro Skin Permeation Test

An in vitro skin permeation study was performed using a Franz diffusion cell to investigate the enhancement in skin permeation of oleanolic acid. The test was carried out on PMO-G, PMO-H, the mixture of oleanolic acid and Tween 80 dispersed in distilled water, and the mixture of oleanolic acid and propylene glycol dispersed in distilled water. The skin of a 6-week-old female hairless mouse was cut into pieces of the required size. Vertical Franz cells were used, and the skin was fixed between the two chambers with its stratum corneum facing upward. 330 μl of the selected formulation was applied on the skin and the Franz cells were covered with parafilm. The receptor was filled with PBS solution (pH 7.4) and ethanol in a 9:1 (v/v) ratio. The receptor solution was refilled with fresh PBS solution at every sampling time. Samples were withdrawn at 2, 4, 6, 8, 10, 20, and 24 h and analyzed by HPLC. After 24 h, the excess formulation remaining on the skin was removed with Kimwipes (Kimberly-Clark professional, NSW, Australia). The skin used in the permeation study was cleaned with PBS solution and the oleanolic acid remaining in the skin was measured by HPLC. All permeation experiments were carried out in triplicate.

### Statistics of the Study

The experiments have been performed in three repeats independently, and the results of this study were reported as mean ± SD. Statistical analysis was verified by independent *t* test, and the value of *p* < 0.05 was considered statistically significant.

### Ampoule Preparation with PMO-H

For the clinical test, PMO-H was used as the test product. PMO-H, purified water, disodium EDTA, allantoin, dipropylene glycol, propanediol, carbomer, and PEG/PPG/polybutylene glycol-8/5/3 glycerin were added and stirred for 10~15 min using a magnetic stirrer, and then potassium hydroxide was added and the mixture stirred further for 5~10 min. Once the ingredients were homogeneously mixed, sodium hyaluronate and beta-glucan were added and stirred for a further 2~5 min, and then phenoxyethanol, propanediol, caprylyl glycol, and ethylhexylglycerin were added and stirred for 2~5 min. The final formula was added to an ampoule as the test product. The ampoule air bubbles were removed using a vacuum dry oven before test use. A control was prepared following the same method for the test product except that oleanolic acid was excluded.

### Human Application Tests

#### Human Skin Irritation Study

A skin patch test of the cosmetic product, which contained PMO-H in an ampoule, was performed on 25 male and female subjects aged from 22 to 56 years who had agreed to participate in a human skin irritation test. Each test substance was dropped onto the upper arm and fixed with a patch. The patch was attached for 24 h and the degree of stimulation was observed by 2 professionals 30 min, 24 h, and 48 h after patch removal according to the criteria of the International Contact Dermatitis Research Group (ICDRG).

#### Clinical Trial for Wrinkle Improvement

Polymeric micelles containing oleanolic acid in an ampoule as final product form were applied around the eyes of 23 female subjects aged from 30 to 65 years who had agreed to participate in wrinkle improvement test. The subjects met the inclusion criteria and none of the exclusion criteria and agreed to participate in the human application test. The double-blind method was followed by both researchers and test subjects. The test was conducted for 8 weeks, and the evaluation was performed every 4 weeks. The double-blind method and random allocation were used. The test product and the control were separately applied randomly to either the left or right side of the face of the same subject. Five parameters were evaluated by optical profilometry using Skin Visiometer SV 700 (Courage + Khazaka electronic GmbH, Cologne, Germany) every 4 weeks for 8 weeks—average roughness (R3) was the primary endpoint, and the four parameters of skin roughness (R1), maximum roughness (R2), smoothness depth (R4), and arithmetic average roughness (R5) were the secondary endpoints. Increases or decreases of the five parameters on the same area of the subject were measured over time, and the average values of increase or decrease of the five parameters were calculated and compared between control and test product treatments. In addition, a visual observation of the skin was made by professionals. Photographs of eye wrinkles were taken using Janus 1 Mark II (PIE Co., Ltd., Suwon, Korea). The criteria used to determine the visual evaluation scores are shown in Fig. S1, and the visual results are presented in Fig. 13. The parameters were compared and analyzed using a paired-samples *t* test at a reliability of 95% [20–25].

## Results and Discussion

### Determination of Solubilizer, Surfactant, and Polymer

**Fig. 2:**
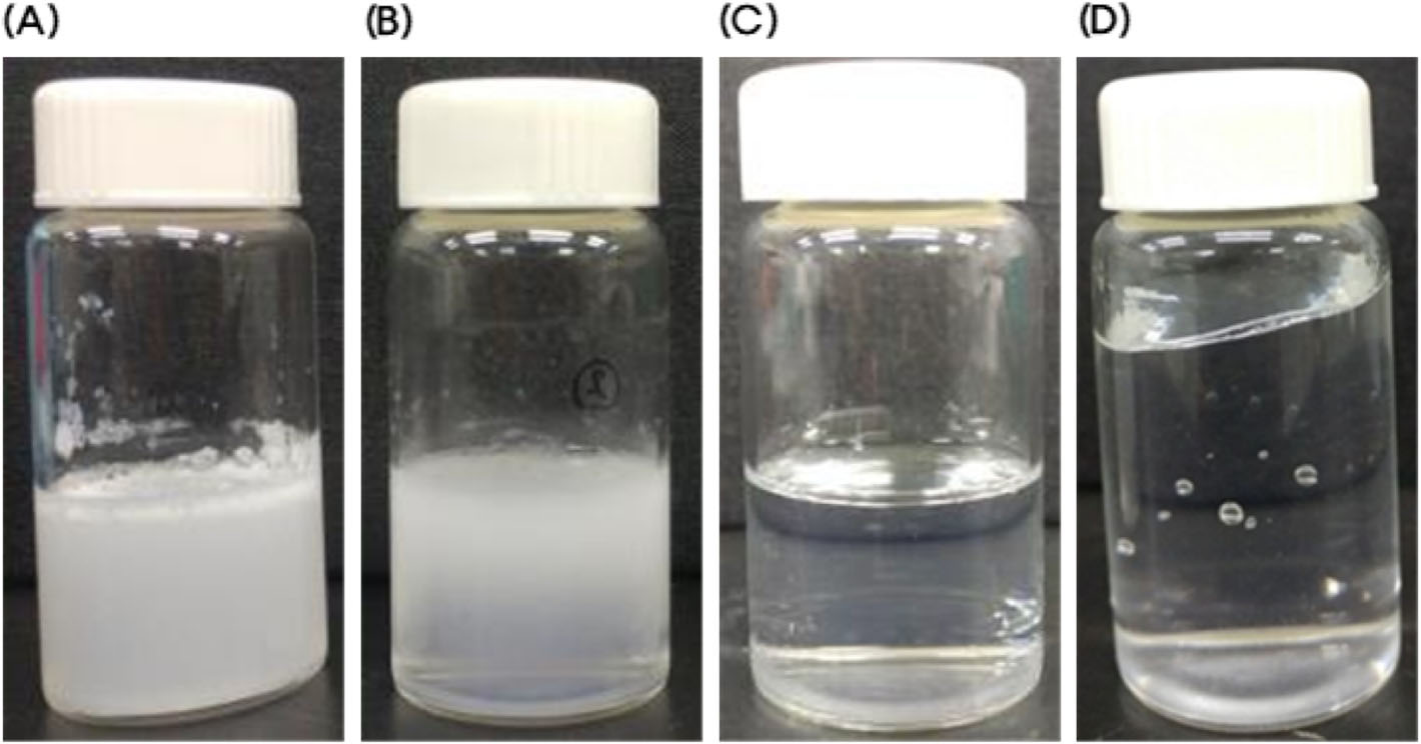
Representative images of **a** precipitation, **b** phase separation, **c** transparent liquid, and **d** gelation of oleanolic acid micelles

The solubility of oleanolic acid in biocompatible solubilizers, specifically surfactants and polymers, are shown in Table 1. Oleanolic acid tends to be soluble in hydrophobic oils with low hydrophilic-lipophilic balance (HLB). Capryol® 90 has a slightly higher HLB value compared to general oils [26]. However, oleanolic acid shows relatively high solubility in Capryol® 90. In addition, Capryol® 90 can be easily stabilized as the inner core of micelles [27]. After selecting Capryol® 90, several types of surfactants or poloxamers were screened for the shell part of the oleanolic acid micelles [27–30]. The oleanolic acid micelle compositions shown in Table 2 are the ones which were transparent immediately after being diluted with distilled water. However, some compositions were unstable and underwent precipitation, phase separation, or gelation within 24 h. Representative images of precipitated, phase separated, transparent liquid, and gel states are shown in Fig. 2. The final state of each formula is summarized in Table 3. Compositions G and H remained transparent even after 24 h. Although composition I remained clear, it was a gel rather than a liquid. Compositions A to D turned opaque with precipitation after 24 h. Given these results, compositions G and H were selected for further development.

### Characteristics of the Polymeric Micelles of Oleanolic Acid

**Fig. 3:**
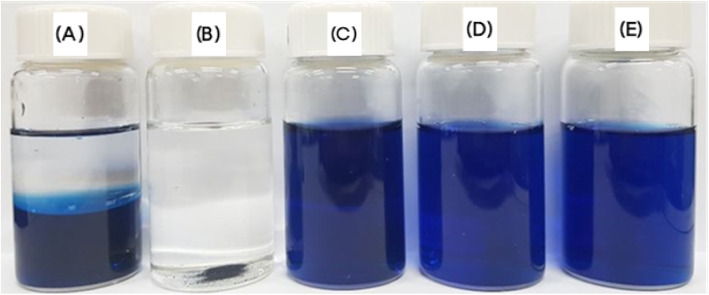
Staining test using methylene blue: (**a**) mixture of Capryol® 90 and distilled water, (**b**) Capryol® 90, (**c**) distilled water, (**d**) PMO-G, and (**e**) PMO-H

The structures of the polymeric micelles of oleanolic acid were investigated by stain test. Figure 3 shows the images of the mixture of Capryol® 90 and distilled water, Capryol® 90, distilled water, and the polymeric micelles of composition G (PMO-G) and of composition H (PMO-H), after adding methylene blue to the solution. Clear phase separation occurred for the mixture of Capryol® 90 and distilled water. Precipitation of methylene blue was observed in the case of Capryol® 90. Distilled water turned dark blue after the addition of methylene blue. PMO-G and PMO-H also turned blue indicating that the polymeric micelles consisted of an oil phase inner core and aqueous phase outer shell. In other words, an amphiphilic polymer, poloxamer 407 in compositions G and H, serves as an outer shell and helps to successfully form polymeric micelles of oleanolic acid by encapsulating the inner core of Capryol® 90 containing oleanolic acid [31].

**Fig. 4:**
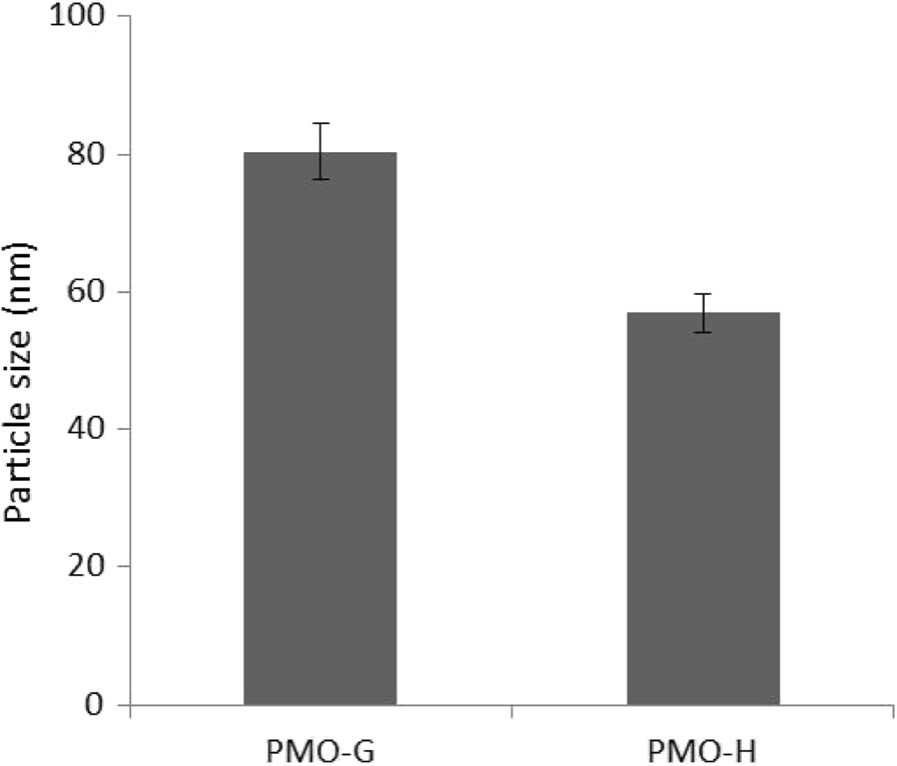
The average particle size of PMO-G and PMO-H from Table 2: 80.4 ± 11.1 nm (PMO-G) and 57 ± 5.24 nm (PMO-H)

**Fig. 5:**
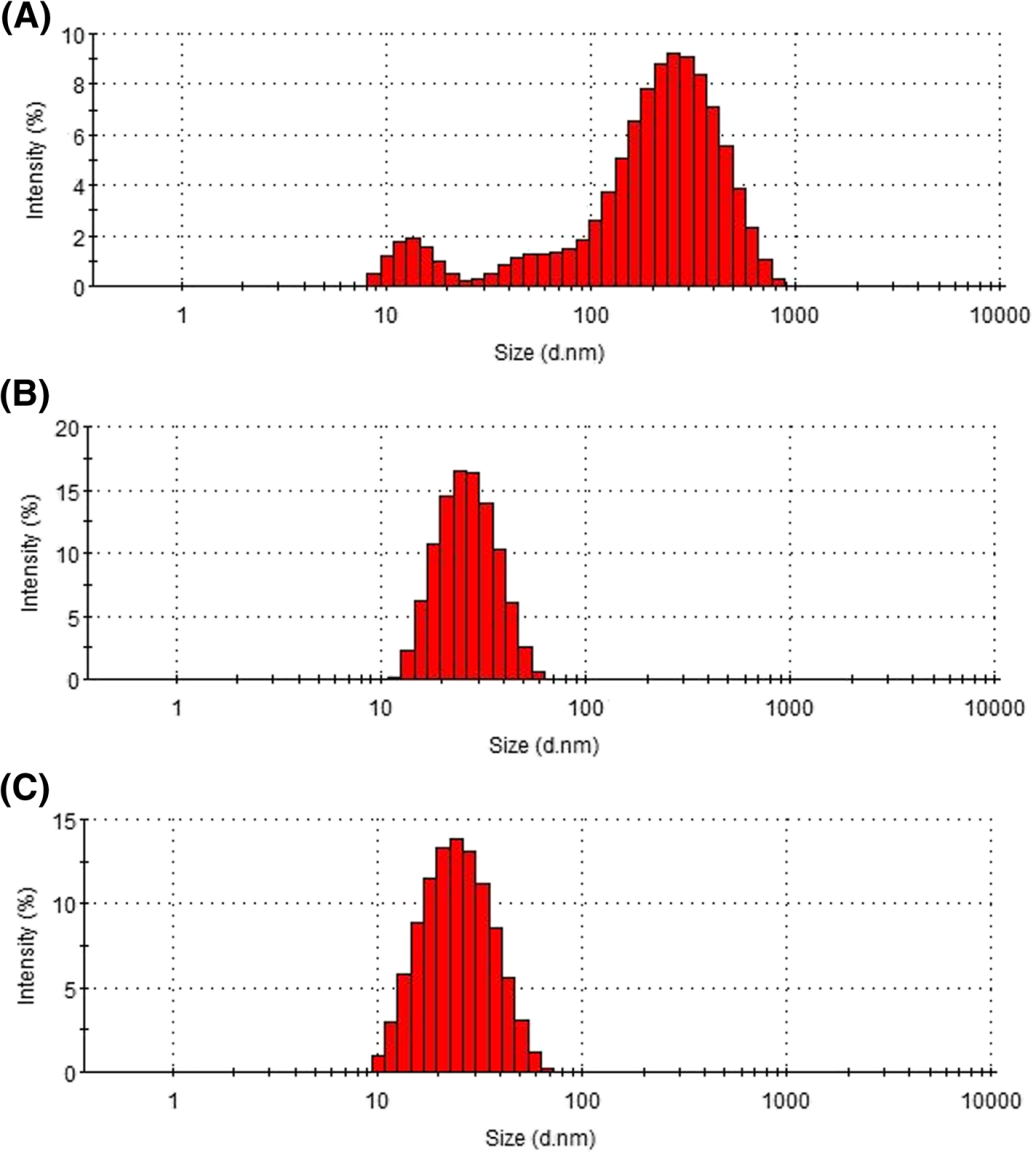
Particle analysis histogram of three different PMO samples. **a** PMO-A, 121.28 nm; **b** PMO-G, 80.4 nm; and (**c**) PMO-H, 57 nm

Particle size, size distribution, and shape can be good indicators for predicting the physical stability of micelle formulations. The average particle size of PMO-G was 80.4 nm, and that of PMO-H was 57 nm (Fig. 4). The histograms of PMO-A, G, and H are shown in Fig. 5 to compare the size distributions affected by phase state. As shown in Table 3, PMO-A is opaque due to precipitation, and PMO-G and H are transparent. The particle size of PMO-A is more than 100 nm, and PMO-A shows a wider size distribution than PMO-G and H (Fig. 5). PMO-H shows a narrower particle size distribution than PMO-G and exhibits a fluidity suitable for cosmetic products.

**Fig. 6:**
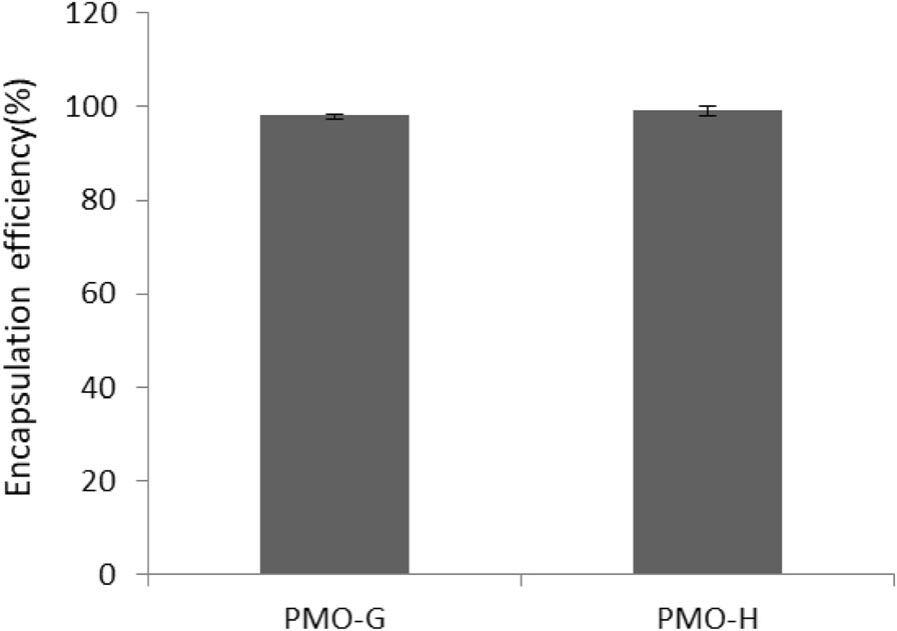
Encapsulation efficacy of PMO-G and PMO-H showing almost 100% drug encapsulation efficiency: 98.26 ± 0.17% (PMO-G) and 99.18 ± 1.06% (PMO-H)

Encapsulation efficiency for both PMO-G and PMO-H was 99 to 100%, indicating that almost 100% of the oleanolic acid was encapsulated in the inner core of PMO (Fig. 6).

**Fig. 7:**
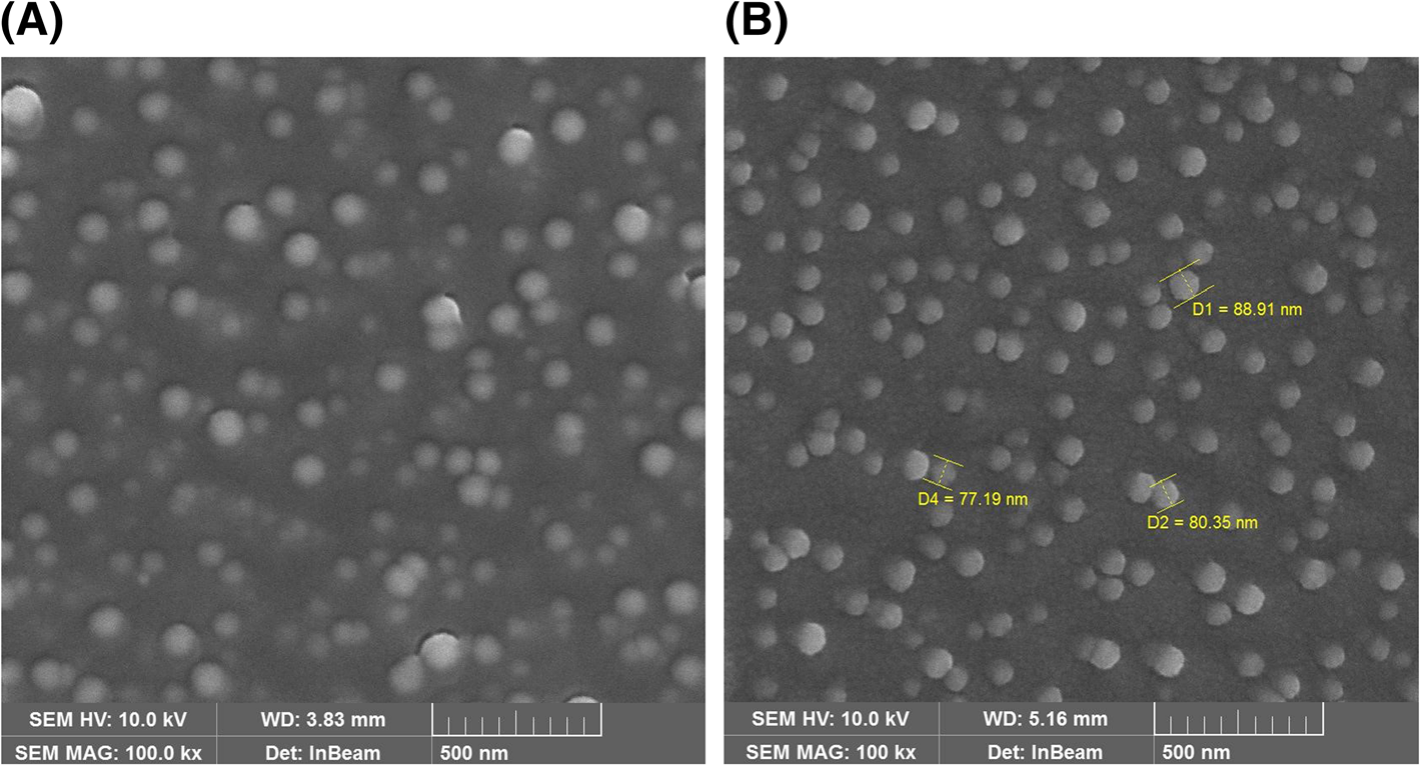
Scanning electron cryomicroscopic images of **a** PMO-G and **b** PMO-H

The shape of PMO-G and PMO-H was investigated by scanning electron cryomicroscopy (Cryo-SEM). Cryo-SEM showed that both PMO-G and PMO-H were spherical shape polymeric micelles. However, PMO-H polymeric micelles were more consistent in size and shape than PMO-G polymeric micelles (Fig. 7).

### In Vitro Skin Permeation Study of PMO

**Fig. 8:**
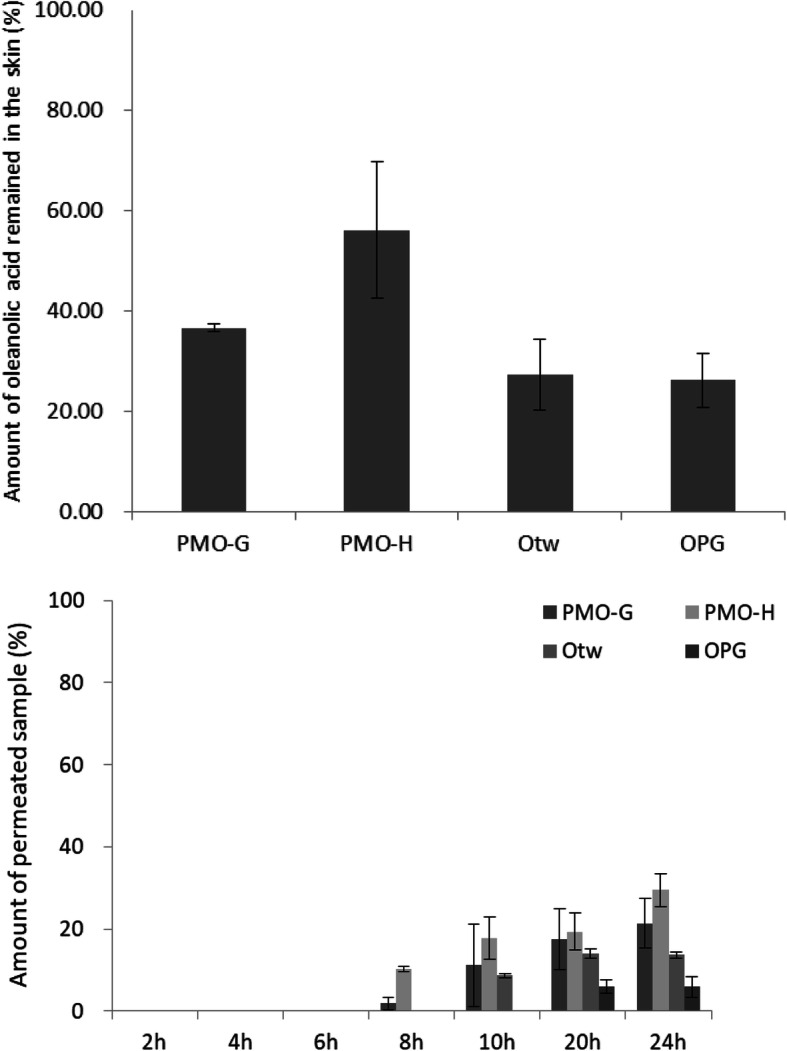
Total amount of oleanolic acid in PMO-G, PMO-H, oleanolic acid, and Tween 80 mixture dispersed in distilled water (Otw) and oleanolic acid and propylene glycol mixture dispersed in distilled water (OPG) permeated through skin and the amount of oleanolic acid in each formulation as a function of time. Amount of oleanolic acid remained in the skin after 24 h: 36.74 ± 0.72% (PMO-G), 56.22 ± 13.50% (PMO-H), 27.44 ± 7.02% (Otw), and 26.28 ± 5.42% (OPG). Amount of oleanolic acid permeated after 24 h: 21.39 ± 5.91% (PMO-G), 29.49 ± 4.00% (PMO-H), 13.66 ± 0.81% (Otw), and 5.90 ± 2.67% (OPG)

The total amount of oleanolic acid remaining in the skin, and the total amount of oleanolic acid permeated through the skin as a function of time were measured using the skin of a 6-week-old female hairless mouse. Four different formulations, PMO-G, PMO-H, the mixture of oleanolic acid and Tween 80 dispersed in distilled water (OTw), and the mixture of oleanolic acid and propylene glycol dispersed in distilled water (OPG) were compared in terms of skin permeation efficiency of oleanolic acid. The total amounts of oleanolic acid permeated through the skin after 24 h were 29.49 ± 4.00% for PMO-H, 21.39 ± 5.91% for PMO-G, 13.66 ± 0.81% for OTw, and 5.90 ± 2.47% for OPG. As shown in Fig. 7, the first detection of oleanolic acid in the case of both PMO-G and PMO-H was possible at 8 h, while the first detection of oleanolic acid in OTw was possible at 10 h, and OPG at 20 h. The proportions of oleanolic acid left in the skin were 56.22 ± 13.50% for PMO-H, 36.74 ± 0.72% for PMO-G, 27.44 ± 7.02% for OTw, and 26.28 ± 5.42% for OPG. PMO-H showed the largest amount of both oleanolic acid permeated through the skin and oleanolic acid remaining in the skin (Fig. 8). These results indicate that PMOs can permeate faster and more than formulations that do not form micelles.

### Stability of Liquid Oleanolic Acid Polymeric Micelles

**Fig. 9:**
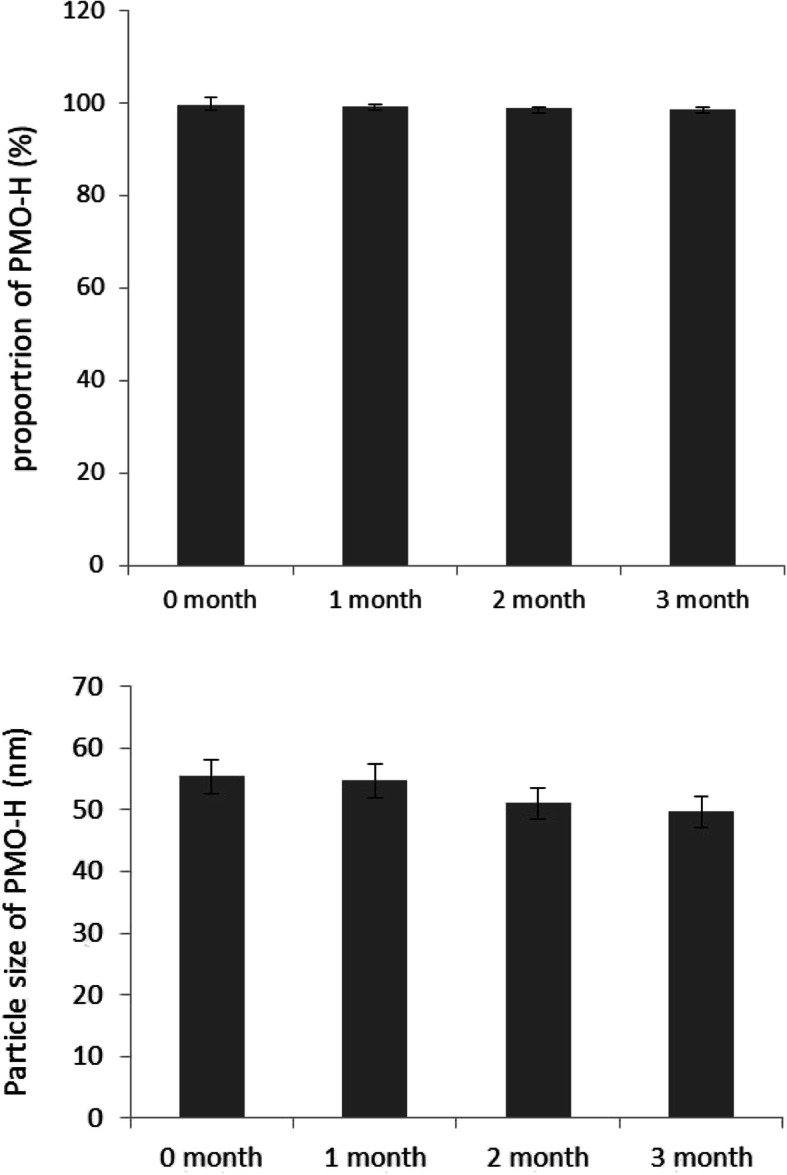
The contents and the particle size changes of PMO-H during 3 months of stability test under acceleration condition at 40 °C/75% RH. Proportion of oleanolic acid in PMO-H: 100 ± 1.6% (0 month), 99.9 ± 0.2% (1 month), 99.5 ± 0.2% (2 month), and 99.3 ± 0.2% (3 month). Particle size of PMO-H: 55.3 ± 6.5 nm (0 months), 54.6 ± 7 nm (1 month), 51.0 ± 5.56 nm (2 months), and 49.6 ± 5 nm (3 months)

On the basis of the characterization and in vitro permeation studies, PMO-H was finally selected for ampoule preparation. Before ampoule preparation, the stability of ONM-H was evaluated. For the stability study, PMO-H was stored in vials under accelerated stability study conditions at 40 °C/75% RH for 3 months. Precipitation, phase separation, color changes, and transparency were then visually evaluated. The proportion of oleanolic acid was then measured using HPLC, and changes in particle size were also checked for. Stability was checked over time. PMO-H remained transparent without precipitation or phase separation, and its color did not change for 3 months under the accelerated stability conditions. The proportion of oleanolic acid as measured by HPLC and the particle size changes over time are shown in Fig. 9. The oleanolic acid proportion remained over 98%, and the particle size of 49.6 ± 5 nm remained almost constant during the 3 month of stability study period. These results show that PMO-H is physically and chemically stable for 3 months under the accelerated stability conditions.

### Clinical Test

#### Human Irritation Test

Before the clinical trial, a human irritation test was conducted on 25 healthy female and male volunteers aged 22~56 years. The test product was patched on the upper arm of the subjects for 24 h, and the skin irritation index was measured 30 min, 24 h, and 48 h after patch removal. Irritation was not observed with the cosmetics ampoule formula containing PMO-H at 1 h or 48 h after patch removal.

### Clinical Trial

The clinical trial was conducted on 23 female subjects, aged 30~65 years with wrinkles around their eyes; excluding the 3 dropouts, 20 subjects completed the trial by separately applying both the test product, the PMO-H ampoule, and the control to either the left or the right sides of their face for 8 weeks. Skin changes were evaluated according to five parameters—average roughness (R3) as a primary endpoint, and four additional parameters as secondary endpoints, namely skin roughness (R1), maximum roughness (R2), smoothness depth (R4), and arithmetic average roughness (R5). The visual evaluation score added a further secondary endpoint. Results are summarized in Table 4.

**Table 4:** Summary of five parameters from clinical trial to evaluated skin wrinkles—an average roughness (R3) as a primary endpoint, and four parameters including skin roughness (R1), maximum roughness (R2), smoothness depth (R4), and arithmetic average roughness (R5) as the secondary endpoints

Evaluation parameter	Time	Average ± standard deviation (A.U)		Rate of change (%)	
		Test product	Control product	Test product	Control product
R1	Before use	0.204 ± 0.063	0.178 ± 0.062	-	-
	4 weeks	0.191 ± 0.053	0.187 ± 0.062	− 4.629	8.037
	8 weeks	0.180 ± 0.056	0.180 ± 0.056	− 9.973	4.799
R2	Before use	0.127 ± 0.033	0.118 ± 0.033	-	-
	4 weeks	0.124 ± 0.029	0.125 ± 0.034	− 1.048	7.261
	8 weeks	0.119 ± 0.034	0.126 ± 0.032	− 5.803	9.536
R3	Before use	0.094 ± 0.023	0.087 ± 0.023	-	-
	4 weeks	0.093 ± 0.023	0.091 ± 0.025	− 0.673	5.127
	8 weeks	0.086 ± 0.020	0.095 ± 0.024	− 7.835	9.971
R4	Before use	0.112 ± 0.037	0.096 ± 0.042	-	-
	4 weeks	0.099 ± 0.030	0.101 ± 0.039	− 8.594	10.764
	8 weeks	0.097 ± 0.036	0.094 ± 0.038	− 9.747	3.491
R5	Before use	0.039 ± 0.017	0.031 ± 0.018	-	-
	4 weeks	0.035 ± 0.015	0.033 ± 0.018	− 6.333	17.556
	8 weeks	0.034 ± 0.014	0.032 ± 0.018	− 7.083	8.556

Rate of change (%) = [(value measured after use − value measured before use)/value before use] × 100

**Fig. 10:**
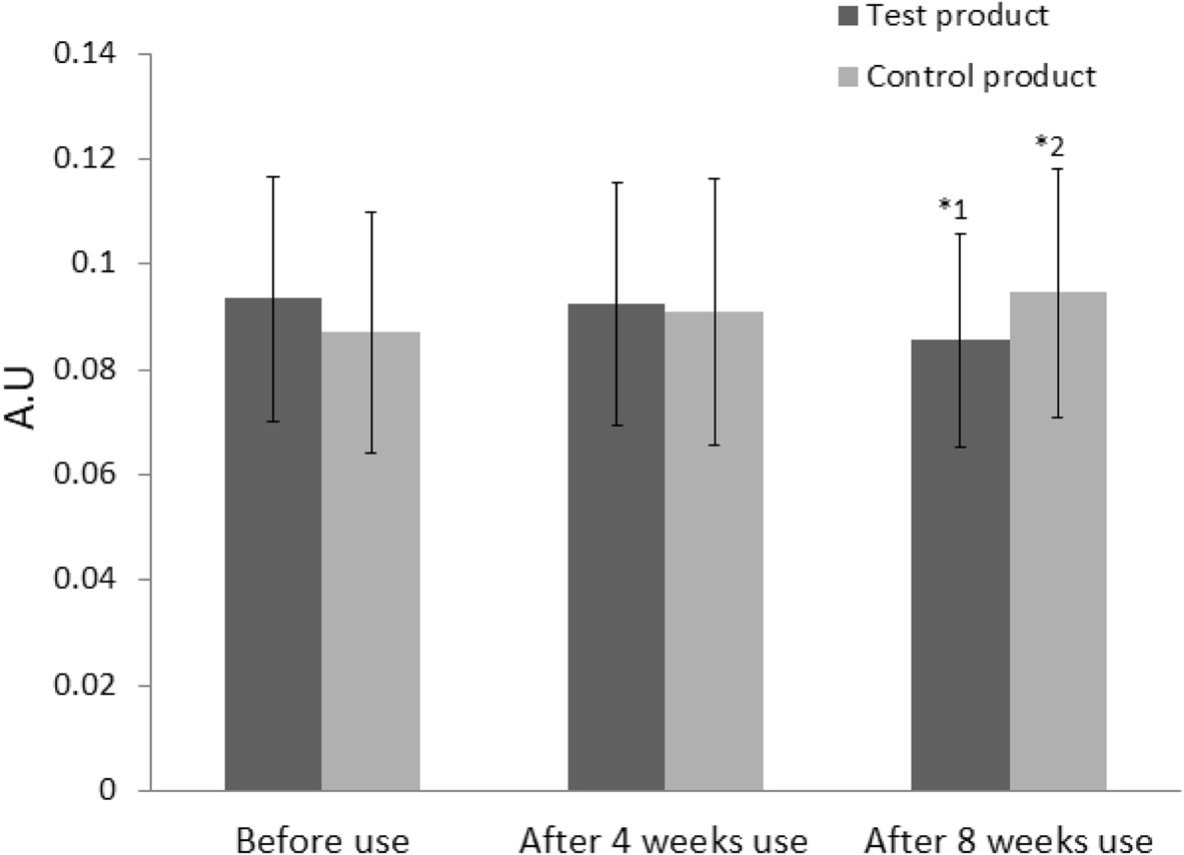
Changes in primary endpoint value, R3, before, after 4 weeks use, and after 8 weeks use of an ampoule containing polymeric micelles of oleanolic acid and the control during clinical trial. R3 value of test product use: 0.094 ± 0.023 before use, 0.093 ± 0.023 after 4 weeks use, 0.086 ± 0.020 after 8 weeks use. R3 value of control product: 0.087 ± 0.023 before use, 0.091 ± 0.025 after 4 weeks use, 0.095 ± 0.024 after 8 weeks use (A.U. for arbitrary unit). *^1^The wrinkle analysis of R3 value decreased statistically significantly. *^2^The wrinkle analysis of R3 value increased statistically significantly

The primary endpoint R3 had decreased by 0.673% after 4 weeks of test product use and statistically significantly by 7.835% after 8 weeks of use (*p* = 0.006). With the control application, R3 had increased by 5.127% after 4 weeks of use and statistically significantly by 9.971% after 8 weeks of use (*p* = 0.010). The difference in R3 value between the areas treated by the test product and those treated by the control was statistically significant after 8 weeks of use (*p* = 0.000) but not statistically significant after 4 weeks of use, perhaps due to inter-subject variation (Fig. 10).

The analysis of the secondary endpoint R1 showed that the value had decreased by 4.629% after 4 weeks of test product use and statistically significantly by 9.973% after 8 weeks of use (*p* = 0.017). With control application, R1 had increased by 8.037% after 4 weeks of use and 4.799% after 8 weeks of use. The difference in R1 values between the areas using the test product and the control was not statistically significant after 4 weeks of use but was after 8 weeks of use (*p* = 0.024). The secondary endpoint R2 had decreased by 1.048% after 4 weeks of test product use and 5.803% after 8 weeks. With control application, it had increased by 7.261% after 4 weeks and 9.536% after 8 weeks. The difference in R2 value between the areas treated by the test product and those treated by the control was statistically significant after 8 weeks of use (*p* = 0.016) but not after 4 weeks of use. The secondary endpoint R4 had significantly decreased by 8.594% (*p* = 0.039) after 4 weeks of test product use and by 9.747% after 8 weeks of use. With the control, R4 had increased by 10.764% after 4 weeks of use and 3.491% after 8 weeks of use. Interestingly, the difference in R4 value between the areas using the test product and the control was statistically significant after 4 weeks of use (*p* = 0.008) but not after 8 weeks. The secondary endpoint R5 had decreased by 6.333% after 4 weeks of test product use and 8.556% after 8 weeks of use. The difference in R5 value between the areas of using the test product and the control was not statistically significant following 4 weeks or even 8 weeks of use.

The analysis of the further secondary endpoint, the visual evaluation of wrinkles, showed that the visual evaluation score had decreased by 2.917% after 4 weeks of test product use and statistically significantly decreased by 8.333% after 8 weeks of use (*p* = 0.034). With application of the control, the visual evaluation score had increased by 1.667% after 4 weeks and 4.167% after 8 weeks. The difference of the visual evaluation score between the areas treated by the test product and by the control was statistically significant after 8 weeks of use (*p* = 0.046) but not after 4.

**Fig. 11:**
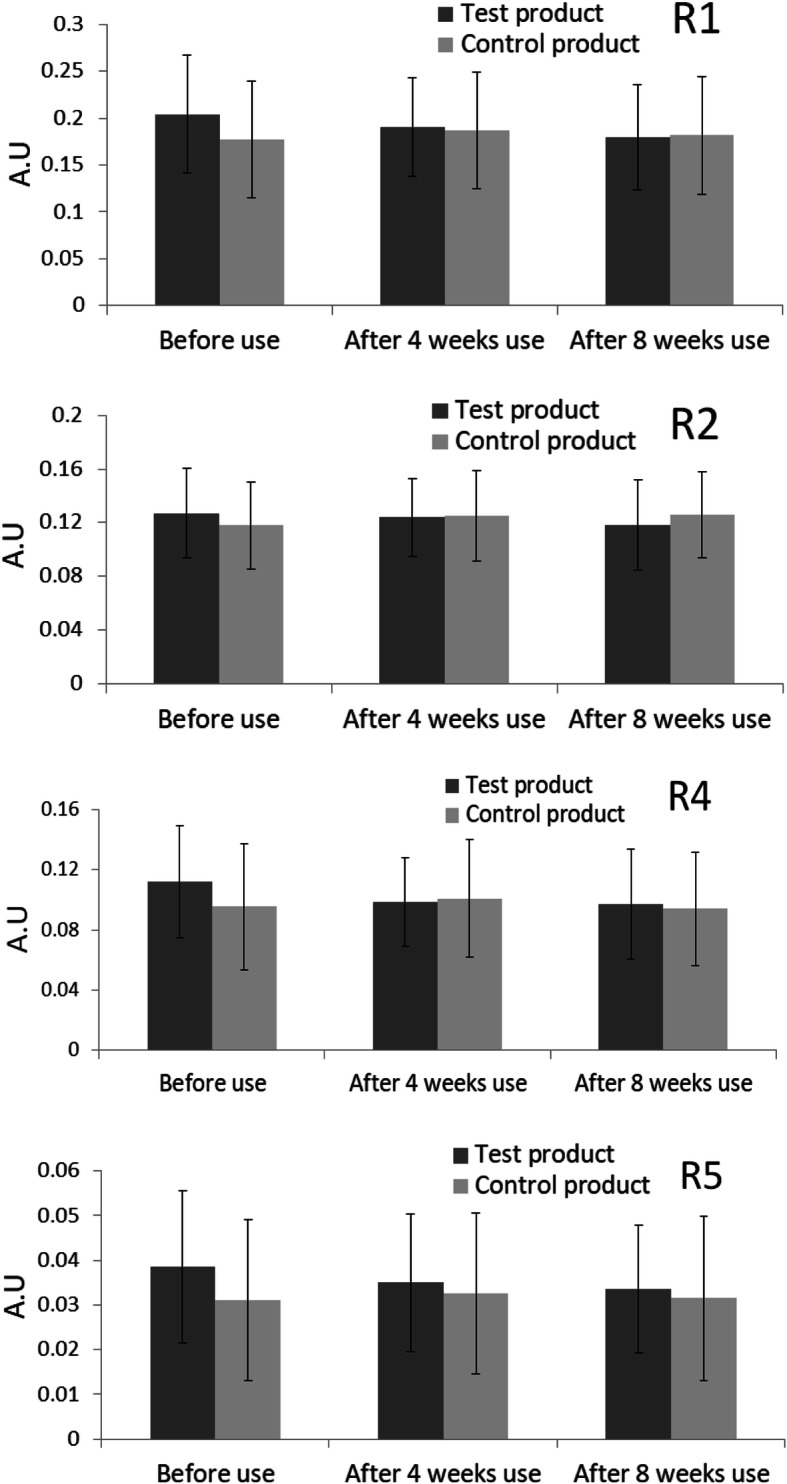
Results of secondary endpoint R1, R2, R4, and R5 for skin wrinkle measurement—before use, after 4 weeks use, and after 8 weeks use of the test product and the control. Please refer to Table 4 for the exact values (A.U. for arbitrary unit)

**Fig. 12:**
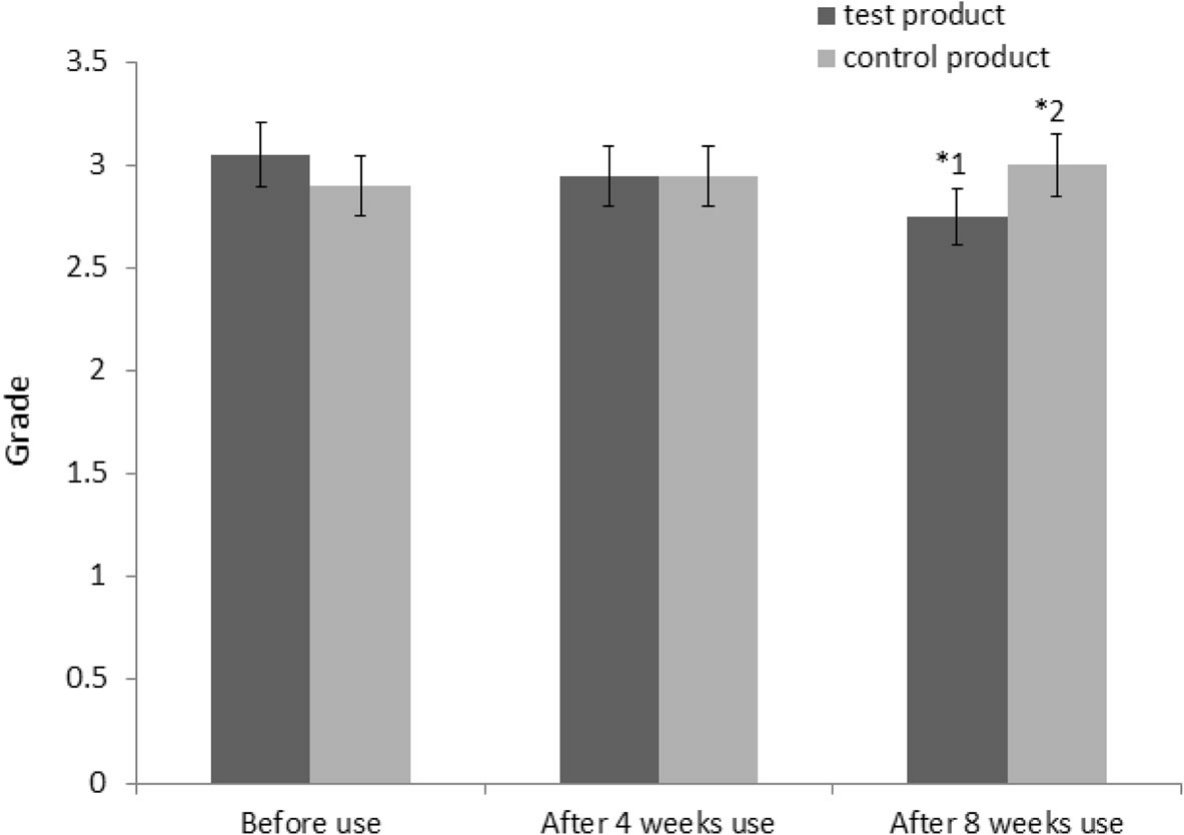
Results of visual evaluation of skin—before use, after 4 weeks use, and after 8 weeks use of the test product and the control. Test product: 3.050 ± 0.887 before use, 2.95 ± 0.999 after 4 weeks use, and 2.75 ± 0.851 after 8 weeks. Control product: 2.900 ± 0.887 before use, 2.95 ± 0.945 after 4 weeks use, and 3.000 ± 0.918 after 8 weeks use. *^1^The visual evaluation grade of wrinkles by professionals decreased significantly. *^2^The visual evaluation grade of wrinkles by professionals increased significantly

**Fig. 13:**
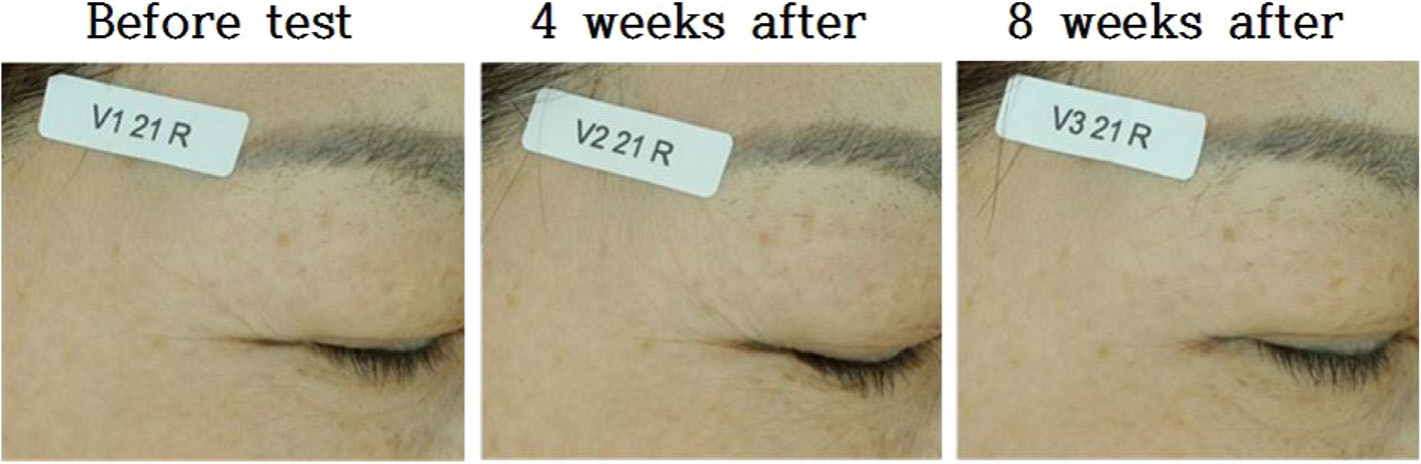
Pictures of the tested areas

In summary, the analysis of the wrinkled area around the eyes showed that the difference in the primary endpoint value R3 between the areas treated by the test product and by the control was statistically significant after 8 weeks of use. In terms of the secondary endpoints, all values had decreased after test product use and increased after control use. The difference in R4 values between the areas treated by the test product and those treated by the control was statistically significant after 4 weeks of use, but the difference of the R1 and R2 values were statistically significant only after 8 weeks of use (Fig. 11). The visual evaluation score by professionals showed that all the average visual evaluation scores for wrinkles had decreased after 4 weeks and 8 weeks of test product treatment compared to the control (Table 5). The difference in the visual evaluation score between the areas treated by the test product and by the control was statistically significant after 8 weeks of use (Fig. 12). Overall, according to all endpoints, the cosmetics formula containing PMO-H as a primary ingredient was found to help improve wrinkles after 8 weeks of use (Fig. 13).

**Table 5:** Summary of visual evaluation scores by professionals—before use, after 4 weeks use, and after 8 weeks use; evaluation parameter are presented in Figure S1

Time	Trial product	Average ± standard deviation (A.U)	Rate of change (%)
Before use	Test product	3.050 ± 0.887	-
	Control product	2.900 ± 0.912	-
After 4 weeks use	Test product	2.950 ± 0.999	− 2.917
	Control product	2.950 ± 0.945	1.667
After 8 weeks use	Test product	2.750 ± 0.851	− 8.333
	Control product	3.000 ± 0.918	4.167

Rate of change (%) = [(value measured after use − value measured before use)/value before use] × 100

## Conclusions

Surfactants are commonly used excipients in cosmetic products to improve solubility of poorly soluble materials. One caveat would be the amount included in the product. Surfactants should be added in sufficient amount to dissolve poorly soluble materials without precipitation. Only a minimal amount should be added for safety reasons. Micelle formulation could be the solution to this problem. Polymeric micelles of oleanolic acid developed in this study improve the solubility of oleanolic acid with a minimum amount of surfactants and enhance the permeation of oleanolic acid through the skin. Stable polymeric micelles of oleanolic acid were developed using Capryol 90 and poloxamer. The polymeric micelles of oleanolic acid developed in this study were stable, that is, they did not show any precipitation, phase separation, or degradation at 40 °C after 3 months. The clinical trial showed that, as a main active ingredient, the oleanolic acid in the polymeric micelle formulation is effective for alleviating human wrinkles. Based on these findings, it can be concluded that oleanolic acid, which is poorly soluble in water and therefore, unusable in a native form as a principal ingredient for alleviating skin wrinkles, can be formulated into applicable polymeric micelles. Furthermore, it is expected that the polymeric micelles of oleanolic acid developed in this study will prove very useful for alleviating human wrinkles and will prove widely applicable to cosmetic applications.

## Supplementary information

**Additional file 1: Figure S1.** Visual evaluation criteria of wrinkles. 0, none; 1, none / mild; 2, mild; 3, mild / moderate; 4, moderate; 5, moderate / severe; 6, severe; and 7, very severe

## Data Availability

Not applicable

## Abbreviations

PMO: Polymeric micelles of oleanolic acid
PEG: Polyethylene glycol
TRANSCUTOL P: Highly purified diethylene glycol monoethyl ether
LABRASOL: PEG-8 Caprylic/Capric Glycerides
LAUROGLYCOL FCC: Propylene glycol monolaurate (type I, monoesters >45%)
LABRAFAC: Caprylic/Capric Triglyceride
Capryol® 90: Propylene glycol monocaprylate (type II, monoesters >90%)
Capryol™ PGMC: Propylene glycol monocaprylate (type I, monoesters >55%)
EDTA: Ethylenediaminetetraacetic Acid
ELS: Electrophoretic light scattering

## References

[1] Giaconomi P (2005). Ageing, science and the cosmetics industry. EMBO Rep.

[2] Lobo V, Patil A, Phatak A, Chandra N (2010). Free radicals, antioxidants and functional foods: impact on human health. Pharmacogn Rev.

[3] Fisher GJ, Wang ZQ, Datta SC, Varani J, Kang S, Voorhees JJ (1997). Pathophysiology of premature skin aging induced by ultraviolet light. N Engl J Med.

[4] Fisher GJ, Kang S, Varani J, Bata-Csorgo Z, Wan Y, Datta S, Voorhees JJ (2002). Mechanisms of photoaging and chronological skin aging. Arch Dermatol.

[5] Zhang K, Lv S, Li X, Feng Y, Li X, Liu L, Li S, Li Y (2013). Preparation, characterization, and in vivo pharmacokinetics of nanostructured lipid carriers loaded with oleanolic acid and gentiopicrin. Int J Nanomedicine.

[6] Moon HI, Chung JH, Lee JK, Zee OP (2004). Triterpenoid saponin from viola hondoensis W. Becker et H Boss. and Their effect on MMP-1 and type Iprocollagen expression. Arch Pharm Res.

[7] Liu J (1995). Pharmacology of oleanolic acid and ursolic acid. J Ethnopharmacol.

[8] Wang MT, Jin Y, Yang YX, Zhao CY, Yang HY, Xu XF, Qin X, Wang ZD, Zhang ZR, Jian YL, Huang Y (2010). In vivo biodistribution, anti-inflammatory, and hepatoprotective effects of liver targeting dexamethasone acetate loaded nanostructured lipid carrier system. Int J Nanomedicine.

[9] Yamamoto K, Nakano M, Arita T, Takayama Y, Nakai Y (1976). Dissolution behavior and bioavailability of phenytoin from a ground mixture with microcrystallin cellulose. J Pharm Sci.

[10] Belhaj N, Arab-Tehrany E, Loing E, Bézivin C (2017). Skin delivery of hydrophilic molecules from liposomes and polysaccharide-coated liposomes. Int J Cosmet Sci.

[11] OECD (2011) Guidance notes on dermal absorption. Series on Testing and Assessment. No. 156.

[12] Leuner C, Dressman J (2000). Improving drug solubility for oral delivery using solid dispersions. Eur J Pharm Biopharm.

[13] Gupta S, Tyagi R, Parmar VS, Sharma SK, Haag R (2012). Polyether based amphiphiles for delivery of active components. Polymer..

[14] Hussein YHA, Youssry M (2018). Polymeric micelles of biodegradable diblock copolymers: enhanced encapsulation of hydrophobic drugs. Materials..

[15] Leibler L, Orland H, Wheeler JC (1983). Theory of critical micelle concentration for solutions of block copolymers. J Chem Phys.

[16] Owen SC, Chan DPY, Shoichet MS (2012). Polymeric micelle stability. Nano Today.

[17] Alvarado HL, Abrego G, Garduño-Ramirez ML, Clares B, García ML, Calpena AC (2015). Development and validation of a high-performance liquidchromatography method for the quantification of ursolic/oleanicacids mixture isolated from Plumeria obtusa. J Chromatogr B Anal Technol Biomed Life Sci.

[18] Xu R (2015). Light scattering: a review of particle characterization applications. Particuology..

[19] Ge HY, Zhao CL, Porzio S, Zhuo L, Davis HT, Scriven LE (2006). Fracture behavior of colloidal polymer particles in fast-frozen suspensions viewed by cryo-SEM. Macromolecules..

[20] Janssen YM, Van HB, Borm PJ, Mossman BT (1993). Cell and tissue responses to oxidative damage. Lab Investig.

[21] Grove GL, Grove MJ, Leyden JJ (1989). Optical profilometry: an objective method for quantification of facial wrinkles. J Am Acad Dermatol.

[22] Griffiths CE, Wang TS, Hamilton TA, Voorhees JJ, Ellis CN (1992). A photonumeric scale for the assessment of cutaneous photodamage. Arch Dermatol.

[23] Grove GL, Grove MJ, Leyden JJ, Lufrano L, Schwab B, Perry BH, Thorne EG (1992). Skin replica analysis of photodamaged skin after therapy with tretinoin emollient cream. J Am Acad Dermatol.

[24] Chung JH, Lee SH, Youn CS, Kim KH, Park KC, Cho KH, Eun HC (2001). Cutaneous photodamage in Koreans influence of sex, sun exposure, smoking, and skin color. Arch Dermatol.

[25] Fu JJJ, Hillebrand GG, Raleigh P, Li J, Marmor MJ, Bertucci V, Grimes PE, Mandy SH, Perez MI, Weinkle SH, Kaczvinsky JR (2010). A randomized, controlled comparative study of the wrinkle reduction benefits of a cosmetic niacinamide/peptide/retinyl propionate product regimen vs. a prescription 0·02% tretinoin product regimen. Br J Dermatol.

[26] Rao SV, Shao J (2008). Self-nanoemulsifying drug delivery systems (SNEDDS) for oral delivery of protein drugs I. Formulation development. Int J Pharm.

[27] Date AA, Nagarsenker MS (2010). Novel delivery systems of atorvastatin should be evaluated for pharmacodynamics instead of pharmacokinetics. J Pharm Pharmacol.

[28] Ghosh CR, Paria S (2012) Core/shell nanoparticles: classes, properties, synthesis mechanisms, characterization, and applications. 112(4):2373-2433. 10.1021/cr100449n 22204603

[29] Rainer H, Müller KM, Gohla S (2000). Solid lipid nanoparticles (SLN) for controlled drug delivery – a review of the state of the art. Eur J Biopharm.

[30] Zhang JL, Srivastava RS, Misra RDK (2007). Core−shell magnetite nanoparticles surface encapsulated with smart stimuli-responsive polymer: synthesis, characterization, and LCST of viable drug-targeting delivery system. Langmuir..

[31] Cammas S, Suzuki K, Sone C, Sakurai Y, Kataoka K, Okano T (1997). Thermo-responsive polymer nanoparticles with a core-shell micelle structure as site-specific drug carriers. J Control Release.

