# Supplementary material for: Development of Polymeric Micelles of Oleanolic Acid and Evaluation of Their Clinical Efficacy
Source: Nanoscale Res Lett. 2020 Jun 22;15:133. doi: 10.1186/s11671-020-03348-3 (PMC7310044; doi:10.1186/s11671-020-03348-3)
Supplement: Supplementary file 1 — Additional file 1: Figure S1. Visual evaluation criteria of wrinkles. 0, none; 1, none / mild; 2, mild; 3, mild / moderate; 4, moderate; 5, moderate / severe; 6, severe; and 7, very severe [file 11671_2020_3348_MOESM1_ESM.docx]

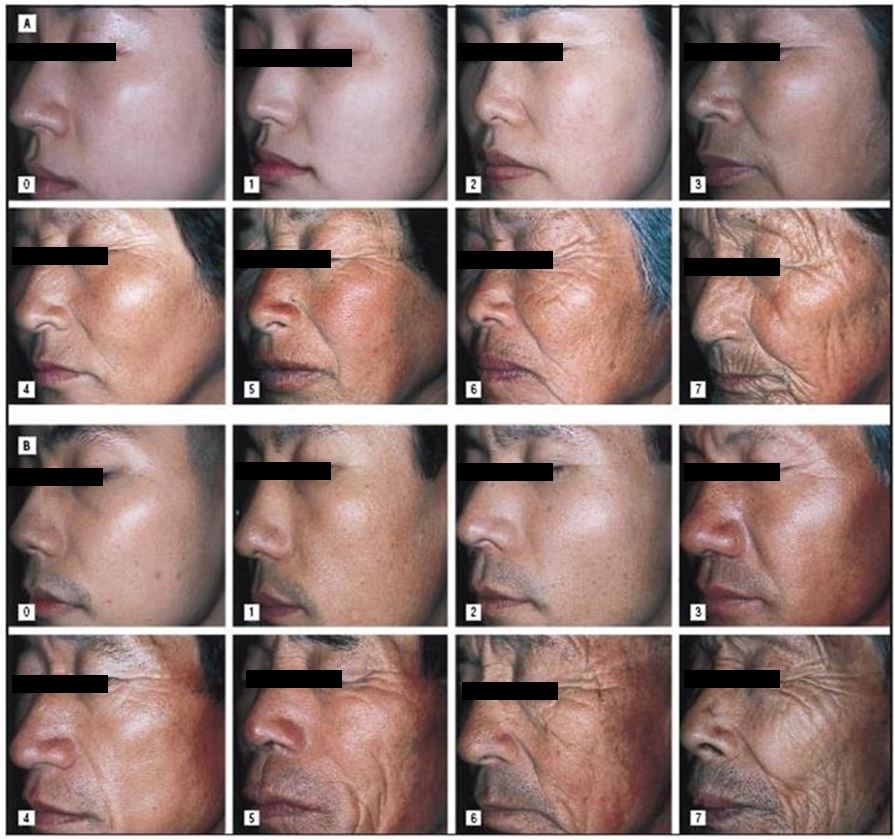


Figure S1. Visual evaluation criteria of wrinkles. 0, none; 1, none ⁄ mild; 2, mild; 3, mild ⁄ moderate; 4, moderate; 5, moderate ⁄ severe; 6, severe; and 7, very severe
